# Experience of Cardiovascular and Cerebrovascular Disease Surgery Patients: Sentiment Analysis Using the Korean Bidirectional Encoder Representations from Transformers (KoBERT) Model

**DOI:** 10.2196/65127

**Published:** 2025-05-30

**Authors:** Hocheol Lee, Yu Seong Hwang, Ye Jun Kim, Yukyung Park, Heui Sug Jo

**Affiliations:** 1 Department of AI Health Information Management, Software Digital Healthcare Convergence College, Yonsei University, Wonju, Republic of Korea; 2 Department of Health Policy and Management, School of Medicine, Kangwon National University, 510 School of Medicine Building #1 (N414), 1, Kangwondaehak-gil, Chuncheon, Gangwon-do, 24341, Republic of Korea, 82 332508910; 3 Division of Data Science, Yonsei University, Wonju, Republic of Korea; 4 Department of Preventive Medicine, Kangwon National University Hospital, Chuncheon, Republic of Korea

**Keywords:** cardiovascular, cerebrovascular, KoBERT, artificial intelligence, TCM, transitional care model, hospitalization, care model, surgery, emotional experiences, emotion, South Korea, Asia, web portal, transformer, sentiment analysis, health status, rehabilitation, caregiver support, cost management, Korean Bidirectional Encoder Representations from Transformers

## Abstract

**Background:**

Cardiovascular and cerebrovascular diseases significantly contribute to global mortality and disability. The shift to outpatient postoperative care, accelerated by the COVID-19 pandemic, emphasizes the need for effective management of postoperative outcomes. The high rates of cardiovascular and cerebrovascular diseases in Korea necessitate focused transitional care during patient discharge periods. However, limited research exists on the postoperative experiences of discharged patients, underscoring the necessity of establishing evidence-based services to optimize transitional care.

**Objective:**

The objective of this paper was to analyze the emotional experiences of patients who underwent cardiovascular and cerebrovascular surgeries using data from Naver, a major South Korean web portal.

**Methods:**

Posts were collected using specific keywords and processed with the Korean Bidirectional Encoder Representations from Transformers (KoBERT) model based on Transformer, which classified sentiments into positive, neutral, and negative categories. Model performance was validated according to precision, recall, *F*_1_-score, and support. Sentiment analysis was conducted within the Transitional Care Model (TCM) framework, divided into 5 domains: health status, care resources, care demand, interaction, and mental state.

**Results:**

The KoBERT model demonstrated high classification performance, achieving a precision of 96%, recall of 94%, and an *F*_1_-score of 94%. Sentiment analysis revealed that compared with cardiovascular surgery patients, cerebrovascular surgery patients experienced higher negative emotions regarding health status, whereas cardiovascular surgery patients expressed more negative sentiments in care demands.

**Conclusions:**

Different patient groups experience distinct emotional and practical challenges postdischarge. Particularly, keywords within the TCM framework highlight that cerebrovascular surgery patients require robust rehabilitation and caregiver support, whereas cardiovascular surgery patients need better cost management. These findings underscore the importance of personalized transitional care strategies tailored for cardiovascular and cerebrovascular diseases. The insights derived from this study can guide health care policymakers in designing more targeted and patient-centered interventions to improve postdischarge care and patient-centered transitional care, ensuring continuous and effective postoperative management.

## Introduction

Cardiovascular and cerebrovascular diseases are among the leading causes of mortality worldwide, causing 17.5 million deaths annually. Ischemic heart disease and stroke rank as the second and fourth leading contributors to the global disease burden, respectively. Furthermore, the disability-adjusted life years (ie, years lost due to premature death and years lived with disability) related to ischemic heart disease and stroke are projected to increase progressively [1,2]. In addition, with increases in life expectancy owing to advancements in medical technology and improved health care access, the number of older patients undergoing cardiac and neurological surgeries also continues to increase [3,4]. Although postoperative prognostic monitoring has traditionally been conducted in hospital settings, the COVID-19 pandemic has accelerated a shift toward outpatient treatment for prognosis, facilitated by advancements in surgical techniques and improved success rates [5]. This transition offers several benefits, including reduced medical costs and a quicker return to normal life. However, it also underscores the need for further research on the management of postoperative outcomes in outpatient settings. There have been reports of patients experiencing deterioration or death owing to inadequate management or reduced continuity of care following a transition from inpatient to outpatient treatment [6,7]. Considering these concerns, patients often experience anxiety and uncertainty regarding the appropriateness of postoperative discharge decisions during transitional periods during transitional periods.

These transitional periods refer to the change among health care providers, medical staff, or the health service environment. The period during which patients are discharged from hospitals to their homes or other facilities is critical. During this time, patients face a higher risk of medical complications, management failures, and adverse drug reactions because of potential interruptions in treatment, care, and information flow [8-10]. “Transitional care services” refer to interventions provided to high-risk patients with multiple vulnerabilities to mitigate risk during critical transition periods and ensure continuous care, enabling them to effectively manage and recover from illnesses [8]. This extends beyond medical management and focuses on providing services tailored to the unique lives and needs of patients with specific conditions.

Numerous studies have demonstrated the health-promoting and economic benefits of transitional care services [11]. The Transitional Care Model (TCM) comprises several core components, namely, screening, staffing, relationship maintenance, patient and family caregiver engagement, risk and symptom assessment and management, education and self-management promotion, collaboration, continuity promotion, and coordination fostering [12]. Extensive research shows that TCM provides a validated structure for analyzing postdischarge challenges by integrating components such as health status monitoring, care resource coordination, patient-provider interaction, and mental health support [13]. Many Organisation for Economic Cooperation and Development (OECD) countries, including the United States with its Hospital Readmission Reduction Program, incentivize hospitals to provide transitional care services to reduce readmissions. However, in Korea, transitional care services remain at the pilot stage and have not yet been implemented for the general public. By analyzing the emotional experiences encountered during the transitional period of patients who have undergone hospitalization and surgery for cardiovascular and cerebrovascular diseases, valuable insights may be gained regarding the need for transitional care services in a patient-centered care design and further improvements in the care system.

Recently, artificial intelligence (AI)–driven sentiment analysis has enhanced personalized health care and improved postoperative care. Through sentiment analysis using large language models (LLMs), patient emotions extracted from clinical records and nursing diaries have provided insights into emotional well-being and its impact on recovery from cardiovascular disease [14]. In addition, LLMs have been used to assist health care providers in developing more patient-centered treatment strategies by identifying sentiment-based patterns in medical documentation [15]. The Bidirectional Encoder Representations from Transformers (BERT) model, introduced in 2018, has shown outstanding capabilities in natural language processing and has been applied to various emotional analyses [16]. This has led to a growing body of research that uses the BERT model for emotional analysis in the health care sector. South Korea is among the most rapidly advancing countries in information technology within the OECD, boasting exceptionally high rates of smartphone adoption and social media engagement [17]. Particularly, patients who have undergone brain or cardiac surgery often share their postoperative progress, physical condition, and psychological well-being with fellow patients who have undergone similar procedures. With South Korea’s advanced digital infrastructure and high levels of internet use, an increasing number of discharged patients share status updates through online support groups or online communities. This information is continuously accumulated as unstructured data on various websites, and there is a growing trend of analyzing these posts to study patients’ emotional states and experiences [18]. However, existing research on the postoperative experiences of patients undergoing cardiovascular and cerebrovascular disease surgery remains limited, leading to a lack of empirical evidence necessary for developing effective transitional care strategies. In particular, comprehensive investigations into the challenges and needs of patients during their recovery period are scarce, emphasizing the urgent need for evidence-based, tailored support services. The objectives of this study were as follows: (1) to analyze the emotional states of patients who have undergone cerebrovascular and cardiovascular surgeries, (2) to categorize these emotional states based on TCM, (3) to identify scores and keywords in each field related to the patients, and (4) to provide policy recommendations for the management of surgical patients based on these findings. Toward this goal, the Korean Bidirectional Encoder Representations from Transformers (KoBERT) model was applied for sentiment analysis of postdischarge cardiovascular and cerebrovascular surgery patients. Data were integrated with the TCM framework to categorize patient emotions across 5 key domains, identify disease-specific emotional trends, and provide practical insights for personalized transitional care and health care policy improvements.

## Methods

### Study Design

**Figure 1.**
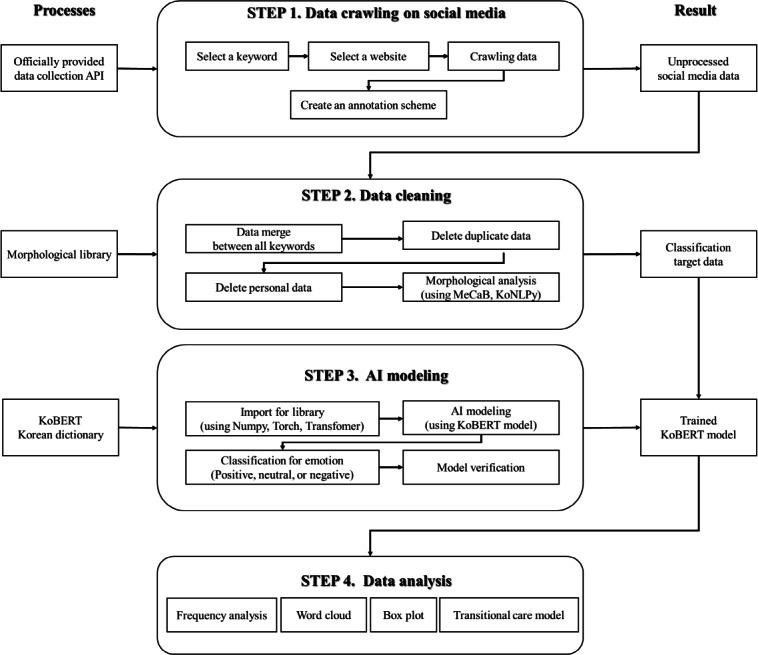
Research framework. API: application programming interface; MeCaB: yet another part-of-speech and morphological analyzer; KoNLPy: Korean natural language processing in Python; AI: artificial intelligence; KoBERT: Korean Bidirectional Encoder Representations from Transformers.

This study investigated the emotional experiences of patients who underwent cerebrovascular or cardiovascular surgery and were subsequently discharged from the hospital. The research methodology used a four-step process: (1) data crawling, (2) data cleaning, (3) AI modeling, and (4) data analysis. Figure 1 shows a schematic of the workflow.

### Ethical Considerations

Institutional review board approval was not sought, as the study did not involve human subjects. All websites were publicly accessible, and only archived versions were reviewed. No identifiable private information was accessed or recorded.

### Data Collection

This study used data collected from Naver [19], South Korea’s premier search engine, and web portals. Naver offers a comprehensive suite of services, including search engines, portals, blogging platforms, discussion forums, and e-commerce solutions, and has the largest user base in Korea. As of November 2024, Naver’s search engine usage rate in South Korea stands at 77.4%, significantly higher than that of Google, the second most used search engine, at 34.1%. In addition, Naver grants crawling application programming interface (API) access exclusively to researchers, whereas other search engines and social media platforms have restrictions on API access. As such, only Naver was included in this study [20]. In addition, it was selected for this study because of its diverse user demographics, particularly the broad age range of the blog contributors. Data were collected using Naver’s API in Python (Python Software Foundation), targeting posts published over the past decade. The keywords used for data crawling included “stroke,” “cerebral hemorrhage,” “cerebrovascular,” “cardiovascular disease,” “surgery review,” “inpatient review,” and “discharge review,” resulting in a total of 10 distinct subsets. Each subset was limited to a maximum of 1000 posts, yielding a comprehensive corpus of 9393 blog posts for analysis (see Table 1).

**Table 1.** Keywords for crawling data.

Patient group and subset		Posts collected (N=9393), n (%)	Crawling keywords	
			Keyword 1	Keyword 2
Cerebrovascular disease				
	1	872 (9.28)	Surgery review	Stroke
	2	990 (10.53)	Surgery review	Cerebral hemorrhage
	3	951 (10.12)	Surgery review	Cerebrovascular
	4	850 (9.05)	Inpatient review	Stroke
	5	970 (10.33)	Inpatient review	Cerebral hemorrhage
	6	930 (9.90)	Discharge review	Stroke
	7	960 (10.22)	Discharge review	Cerebral hemorrhage
	8	910 (9.69)	Discharge review	Cerebrovascular
Cardiovascular disease				
	9	1000 (10.65)	Surgery review	Cardiovascular
	10	960 (10.22)	Discharge review	Cardiovascular

### Data Cleaning

The collected data were preprocessed for analysis. All posts were initially consolidated into a single dataset (data merging), and then 787 duplicate posts were subsequently eliminated along with posts related to animals. Redundant content was removed, and personal information (eg, names, phone numbers, and addresses) was redacted. Preprocessing was conducted using a MeCab morphological analyzer from the Korean Natural Language Processing in Python library. MeCab was selected for its high accuracy, efficient part-of-speech recognition, high processing speed, multilingual support, and ease of installation. From the 9393 posts, 575,505 words were extracted for analysis.

### Data Training

For the sentiment analysis of crawled posts, this study used the KoBERT model. Briefly, KoBERT is a deep learning model for Korean natural language processing and is based on Google’s BERT model. It is widely used in applications such as chatbots, search engines, and machine translations. In this study, the model was trained using the Korean Sentiment Dictionary from the Emotional Dialogue Corpus. This corpus is a comprehensive natural-language sentiment dictionary comprising 15,700 sentences from 1500 Korean speakers and 270,000 sentences encompassing 60 detailed sentences [21]. The Emotional Dialogue Corpus served as a sentiment dictionary for training the KoBERT model. For model training, the dataset was split into 216,000 samples (80%) for the training set and 54,000 samples (20%) for the test set (see Table 2).

The KoBERT model was trained to classify the sentiment of the data (posts) into 3 sentiment score categories: “positive,” “neutral,” and “negative.” Relevant documentation is available on GitHub [22]. We used KoBERT as the base model for sentiment classification. The input text was first tokenized using the MeCab morphological analyzer with a maximum sequence length of 64 tokens. The tokenized input was then processed through the KoBERT transformer encoder, followed by a fully connected classification layer to predict the sentiment as positive, neutral, or negative. For training, we used the Adam optimizer with a learning rate of 5×10^-5^, a warmup ratio of 0.1, and gradient clipping with a max norm of 1 to stabilize learning. The model was trained for 3 epochs with a batch size of 64. To evaluate computational performance, the training and inference times were measured. The total training time per epoch was approximately 4.15 minutes, while the inference time per batch was approximately 233 ms. A summary table of hyperparameters and a diagram illustrating the model architecture are included for clarity.

**Table 2.** Model training and analysis parameters.

Parameter	Value	Description	
Model	KoBERT^a^	Korean Bidirectional Encoder Representations from Transformers–based sentiment classification model.	
Pretrained dataset	Korean Sentiment Corpus	Used for initial model training.	
Optimizer	Adam	Optimizer for gradient descent.	
Learning rate	5×10^-5^	Initial learning rate for model training.	
Log_interval	200	Gradient clipping value.	
Batch size, n	64	Number of samples per batch	
Epochs, n	3	Number of training iterations.	
Max_len, n	64	Maximum token length per input text.	
Max_Grad_norm	1	Logging interval during training.	
Train split, n (%)	216,000 (80)	Rate of train set.	
Test split, n (%)	54,000 (20)	Rate of test set.	
Evaluation metrics	Precision, recall, and *F*_1_-score	Performance measures for sentiment classification.	

a KoBERT: Korean Bidirectional Encoder Representations from Transformers.

### Data Validation

The performance of the AI deep learning model was assessed according to 4 key metrics: precision, recall, *F*_1_-score, and support. The operational definitions of these metrics are provided in the subsections below.

#### Precision

Precision indicated the percentage of actual positive samples among cases predicted to be positive by the machine learning (ML) model. Particularly, it referred to the percentage of the actual sentiment classes relative to the predicted sentiment classes. Precision was calculated as follows:


$$Precision\;\left(\%\right)=\left(\frac{t_{p}}{t_{p}+f_{p}}\right)\times 100$$

#### Recall

Recall showed the percentage of sentiment classes predicted by the ML models relative to the actual sentiment classes. Specifically, it is the percentage predicted to show the risk group among the actual sentiment classes. Recall was calculated as follows:


$$Recall\;\left(\%\right)=\left(\frac{t_{p}}{t_{p}+f_{n}}\right)\times 100$$

#### *F*_1_-score

The *F*_1_-score represented the harmonic mean of precision and recall. The mathematical formula was as follows:


$$F1score\left(\%\right)=\left(\frac{2t_{p}}{2t_{p}+f_{p}+f_{n}}\right)\times 100$$

#### Support

Support represented the number of occurrences of each class in an actual dataset. It was not included in the calculation of any score but provided additional information about the distribution of classes in the dataset. Support is often used to identify class imbalance.

### Data Analysis

Sentiment analysis was performed based on the data collected using the KoBERT model, and a more detailed analysis was conducted using the TCM framework divided into 5 domains. The TCM framework was used as the analytical framework and was restructured from the patient’s perspective into the following 5 domains: health status, care resources, care demand, interaction (with medical staff), and mental state. Detailed information is provided in Multimedia Appendix 1. A detailed analysis of the following 4 focus areas was conducted. First, word clouds were generated to analyze keywords related to the emotions of patients discharged after heart and brain surgery. Second, the postdischarge sentiment scores (calculated as percentages) of the patients who underwent cardiovascular and cerebrovascular surgeries were classified into 3 score categories: positive, neutral, or negative. Each category is presented with its minimum, maximum, 95% CI, median, mean, and variance. Third, patient distribution according to the 5 domains of patient-centered TCM was identified. Finally, the “positive” score category for the emotional state was presented as 5 mean scores (%) based on the TCM framework and presented as box plots. These plots displayed the frequency, quartiles, and minimum and maximum values. All analyses were conducted using GraphPad Prism 10.2.2.

## Results

### Model Verification

The AI KoBERT model used in this study was trained using the Korean Sentiment Dictionary derived from the Emotional Dialogue Corpus. The trained model achieved satisfactory performance. The precision, recall, and *F*_1_-scores exceeded 0.8, indicating a high level of accuracy and reliability in sentiment classification (see Table 3).

**Table 3.** Korean Bidirectional Encoder Representations from Transformers (KoBERT) model verification.

Class	Precision	Recall	*F*_1_-score	Support, n
Positive	0.96	0.89	0.92	1217
Neutral	0.93	0.93	0.93	318
Negative	0.98	0.99	0.98	6054
Average	0.96	0.94	0.94	2529

### Word Cloud

Data pertaining to cerebrovascular and cardiovascular surgeries were visualized and analyzed using word clouds (Multimedia Appendix 2). For cerebrovascular surgery patients, the prominent keywords were “surgery,” “hospital,” “test,” “receive,” and “symptom.” For cardiovascular surgery patients, the key terms that emerged were “surgery,” “hospital,” “receive,” “eat,” “admission,” and “symptom.”

### Sentiment Scores by Patient Type

The patients’ emotional states were categorized as positive, neutral, or negative, and their respective distributions were analyzed. The results revealed a lower proportion of patients with “neutral” emotional state among cerebrovascular surgery patients than among cardiovascular surgery patients (62,896/84,100,74.8% vs 12,456/16,000, 77.9%). Conversely, the proportion of patients with “positive” (16,839/84,100, 20.0% vs 3072/16,000, 19.2%) and “negative” (4364/84,100, 5.2% vs 471/16,000, 2.9%) emotional states was higher among cerebrovascular surgery patients than their cardiovascular surgery counterparts (see Table 4).

**Table 4.** Rates of emotions by patient type.

Patient type and emotional state		Min, %	Max, %	Mean (SD), %		Variance	Range
Total							
	Positive	0	68.8	19.9 (11)		121.0	68.8
	Neutral	0	36.4	4.8 (4.9)		23.9	36.4
	Negative	25	100	75.3 (12)		143.9	75.0
Cerebrovascular							
	Positive	0	68.8	20 (11.2)		126.3	68.8
	Neutral	25	100	74.8 (12.3)		150.7	75.0
	Negative	0	36.4	5.2 (5)		25.2	36.4
Cardiovascular							
	Positive	0	53.3	19.2 (9.7)		93.3	53.3
	Neutral	40	100	77.9 (10)		100.8	60.0
	Negative	0	24	2.9 (3.6)		13.3	24.0

### Keyword Analysis Related to the Transitional Care Model

We analyzed 575,505 words from 9393 posts. The data were classified into 5 domains within the TCM framework and restructured based on the patient’s perspective (see Table 5). For cerebrovascular surgery patients, the top 3 keywords in each domain were as follows: (1) “health status:” sick (n=1860), symptom (n=1819), and sleep disturbance (n=881); (2) “care resources:” mom (n=1792), transfer (n=1774), and caregiver (n=890); (3) “care demand:” rehab (n=1323), postcare (n=689), and check (n=647); (4) “interaction:” hospital (n=5623), test (n=4215), and therapy (n=3117); and (5) “mental state:” good (n=2406), confirm (n=828), and tough (n=716). For cardiovascular surgery patients, the top 3 keywords in each domain were as follows: (1) “health status:” ache (n=410), symptom (n=297), and sick (n=294); (2) “care resources:” counseling (n=205), visit (n=125), and cost (n=104); (3) “care demand:” medication (n=193), preparation (n=108), and check (n=108); (4) “interaction:” hospital (n=898), procedure (n=321), and doctor (n=223); and (5) “mental state:” void (n=625), good (n=410), and confirm (n=313).

**Table 5.** Keywords ranked by occurrence in each component of the Transitional Care Model (TCM) for cerebrovascular and cardiovascular surgery patients.

Rank	Cerebrovascular surgery patients (n=483,922)					Cardiovascular surgery patients (n=75,765)				
	Health status^a^	Care resource^a^	Care demand^a^	Interaction^a^	Mental state^a^	Health status^a^	Care resources^a^	Care demand^a^	Interaction^a^	Mental state^a^
1	Sick (1860)	Mom (1792)	Rehab (1323)	Hospital (5623)	Good (2406)	Ache (410)	Counseling (205)	Medication (193)	Hospital (898)	Void (625)
2	Symptom (1819)	Transfer (1774)	Post-care (689)	Test (4215)	Confirm (828)	Symptom (297)	Visit (125)	Prep (109)	Procedure (321)	good (410)
3	Sleep disturbance (881)	Caregiver (890)	Check (647)	Therapy (3117)	Tough (716)	Sick (294)	Cost (104)	Check (108)	Doctor (223)	Confirm (313)
4	Not sure (878)	Ambulance (844)	Cause (558)	Procedure (1592)	Worry (698)	Side effect (252)	Insurance (94)	Post-care (98)	Teacher (137)	Problem (178)
5	Severe (669)	Visit (645)	Prep (501)	Teacher (1427)	Problem (687)	Severe (211)	Transfer (91)	Workout (94)	Diagnosis (130)	Worry (176)
6	Recovery (650)	Aid (598)	Counseling (443)	Consult (1421)	Prayer (607)	Swelling (185)	Mom (87)	Control (75)	Decision (130)	Stress (128)
7	Alright (622)	Cost (510)	Medication (426)	Professor (1301)	Gratitude (576)	Inflammation (133)	Caregiver (86)	Cause (62)	Prescription (104)	Tough (103)
8	Headache (593)	Insurance (469)	Disability (424)	Nurse (1174)	Scary (407)	Discomfort (113)	Provide (67)	Living (62)	Professor (104)	Prayer (58)
9	Breathing (584)	Counseling (443)	Control (205)	Doctor (887)	Relaxed (349)	Complication (86)	Family (53)	Recovery (59)	Nurse (103)	Complex (57)
10	Side effect (581)	Care (390)	Recovery (127)	Explanation (726)	Tough (286)	Breathing (83)	Premium (23)	Safety (59)	Specialist (82)	Relaxed (49)

a Keyword (n)

### Sentiment Scores by Patient Type

**Figure 2.**
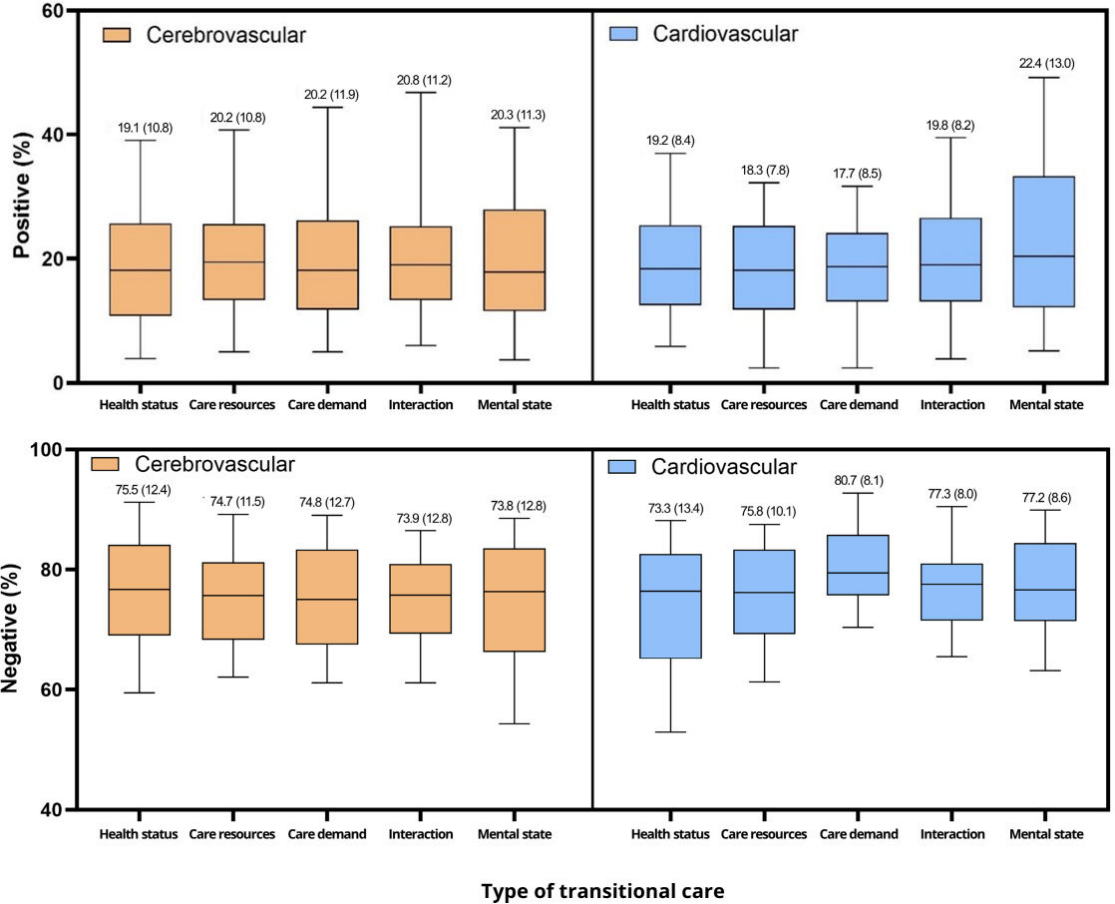
Box plot of sentiment scores by patient type; values are mean (SD).

Figure 2 shows the box plots of the distribution of the sentiment scores (%) for the 5 domains within the TCM framework (health status, care resources, care demands, patient-provider interactions, and mental state).

Compared with cardiovascular surgery patients, cerebrovascular surgery patients demonstrated higher mean postdischarge positive sentiment scores in 3 domains: care resources (3,765/18,600, 20.2% vs 292/1600, 18.3%), care demand (4465/22,100, 20.2% vs 1280/7200, 17.7%), and interaction (2107/10,100, 20.8% vs 575/2900, 19.8%). Conversely, cardiovascular surgery patients exhibited higher mean postdischarge positive sentiment scores in 2 domains: health status (267/1400, 19.2% vs 4467/23,300, 19.1%) and mental state (674/3000, 22.4% vs 2034/10,000, 20.3%). With respect to negative sentiment scores, there was a higher level of dissatisfaction with their health status among patients with cerebrovascular disease than among patients with cardiovascular disease (17,594/23,300, 75.5% vs 2199/3000, 73.3%). Meanwhile, compared with their cerebrovascular counterparts, patients with cardiovascular disease expressed more negative emotions regarding care resources (2198/2900, 75.8% vs 13,898/18,600, 74.7%), care demand (5817/7200, 80.7% vs 16,551/22,100, 74.8%), interactions (1236/1600, 77.3% vs 7467/10,100, 73.9%), and mental state (2316/3000, 77.2% vs 7384/10,000, 73.8%).

## Discussion

### Principal Findings

This study addresses the critical gap in understanding the postoperative experiences of patients who have undergone cardiovascular and cerebrovascular disease surgery. The sentiment analysis revealed significant challenges faced by patients during their recovery, underscoring the need for evidence-based, tailored support services to enhance transitional care strategies. The KoBERT model used for sentiment analysis demonstrated high performance, achieving a precision of 0.96, recall of 0.94, and *F*_1_-score of 0.94.

These results are consistent with those of previous studies that used KoBERT. For instance, a BERT model trained on clinical notes from Korean doctors achieved a precision of 0.99, recall of 0.97, and *F*_1_-score of 0.93. Another KoBERT model trained on sentiments from Korean Internet posts showed precision, recall, and *F*_1_-scores of 0.86 across all metrics. Previous studies have evaluated models using mean squared error, root mean squared error, mean absolute error, and *R*². However, this study focused on classification rather than continuous variables, and thus, these metrics were not used [23].

The performance level of the KoBERT model used in this study is closely similar with that of existing models [24,25], validating its effectiveness in classifying sentiments as positive, neutral, or negative. Furthermore, the model’s capability to identify emotional trends in real time during consultations presents opportunities for its expanded use in relevant research fields. The analysis revealed that the proportion of patients who experienced more negative emotions was higher among those who had underwent cerebrovascular surgery (5.2%) than among those who had undergone cardiovascular surgery (2.9%). This finding is consistent with previous research demonstrating that patients with cerebrovascular disease tend to experience higher levels of anxiety and worry relative to those with other diseases [26]. In a study involving 3200 surgical patients, 40.5% experienced negative postoperative emotions, such as depression, anxiety, and fear. The lower levels of negative emotions observed in our study may be attributed to various factors, including advanced medical technology in Korea leading to high surgical success rates, improved patient management owing to high medical accessibility, and a relatively low financial burden owing to comprehensive health insurance coverage [27-29]. However, it is important to note that the data in this study were obtained by crawling internet posts made by patients after surgery. That is, the patients had access to the internet, potentially indicating a lesser severity of illness, younger age, and relatively good overall physical health. Furthermore, the lower prevalence of negative emotions among cardiovascular surgery patients than among cerebrovascular surgery patients may be explained by the nature of cardiovascular disease treatment in Korea. Most hospitalizations and surgeries related to cardiovascular issues involve ischemic heart disease, which is often treated with percutaneous coronary intervention rather than major surgery. This less invasive approach typically leads to quicker discharge and a lower risk of long-term disability in cardiovascular diseases than in cerebrovascular diseases [30].

TCM is designed to provide continuous care for patients transitioning postoperatively from the hospital to home or another care facility. This study analyzed keywords and sentiment scores (calculated as percentages) across 5 TCM domains classified from the patient’s perspective: health status, care resources, care demand, interaction, and mental state. Across all conditions, keywords indicating physical pain and side effects, such as pain, sickness, and severe, were frequently mentioned in relation to health status. Regarding mental state, negative emotional keywords, such as tough, worry, and problems, were frequently mentioned. This highlights the impact of hospitalization and surgery on patients with severe conditions, such as cardiovascular and cerebrovascular diseases, as well as the need for related care. The interaction-related keywords mainly involved medical care and treatment, including hospitals, procedures, and doctors. This highlights the absence of elements crucial for transitional care services in the Korean health care setting, such as coordination, education, consultation, and explanations. Disease-specific differences were also observed, especially in the domains of care resources and demand, indicating that postdischarge care resources and needs varied according to disease type. For patients with cerebrovascular disease, the highlighted keywords were mother, caregiver, transfer, ambulance (care resources), rehabilitation, and postcare (care demand). This indicated a high demand for postsurgery or postdischarge rehabilitation and related transfers. In contrast, for patients with cardiovascular diseases, the keywords were insurance and cost (care resources) and medication and workout (care demand), indicating a high demand for cost-related items. These disease-specific differences in TCM domain keywords reflect varying patient needs during transition periods. This information is crucial and should be incorporated when modeling patient-centered transitional care services.

When comparing the sentiment scores of patient-centered TCM domains for cardiovascular and cerebrovascular diseases, the positive score categories for health status were similar, accounting for 19.1% and 19.2% of the total scores, respectively. However, in the domains of care resources, care demand, and interaction, the cerebrovascular surgery patients exhibited higher positive sentiment scores than did the cardiovascular surgery patients. This disparity may be due to the fact that cerebrovascular diseases often involve longer postsurgery or postdischarge processes and are more likely to involve additional care services such as rehabilitation, leading to more positive keywords being mentioned during the recovery process. Regarding mental state, cardiovascular surgery patients showed a higher positive score than did cerebrovascular surgery patients (22.4% vs 20.3%). This difference may be attributed to the typically shorter hospital stay of cardiovascular surgery patients and the fact that their immediate problems are often resolved through procedures. These factors can potentially contribute to a more positive average sentiment score among cardiovascular surgery patients than among cerebrovascular surgery patients.

For cerebrovascular diseases, the positive sentiment score category related to interaction (20.8%) was notably higher than that related to other areas, with health status scoring the lowest (19.1%). In contrast, for cardiovascular diseases, the positive sentiment score category was the highest for mental state (22.4%), whereas it was the lowest for care demand (17.7%). These findings may be attributed to the nature of cerebrovascular diseases in which prolonged interactions with medical staff, rehabilitation specialists, and caregivers are crucial during the transition period. Conversely, for cardiovascular diseases, despite the recognized importance of postdischarge cardiac rehabilitation, it appears to be underdeveloped, as reflected by lower scores. This finding is consistent with previous findings showing that only 44.2% of stroke patients discharged from university hospitals continue outpatient rehabilitation and that only 17% of cardiovascular surgery patients discharged from regional cardiovascular centers pursue outpatient cardiac rehabilitation [31,32]. This analysis of sentiment scores for each disease type suggests which components of transitional care are lacking and require improvement, as determined based on patients’ reported experiences that highlight positive or negative sentiments.

Negative sentiment analysis revealed the highest score for health status (75.5%) for cerebrovascular diseases, surpassing that for cardiovascular diseases. This finding aligns with the lower positive sentiment score in this category, likely because of the high risk of postdischarge sequelae. Postdischarge sequelae are closely associated with physical and mental health challenges and result in increased negative sentiment scores. Conversely, among cardiovascular surgery patients, negative sentiment scores were the highest for care demands (80.7%), followed by those for care resources (75.8%), interactions (77.3%), and mental state (77.2%). These scores were higher than those among cerebrovascular surgery patients. This suggests that although cardiovascular diseases significantly affect patient lives, the relatively underdeveloped infrastructure and resources for postdischarge transitional care services may still contribute to the higher negative scores. Patients experience an ongoing need for care, such as cardiac rehabilitation, which can be burdensome and may exacerbate negative sentiments.

Consequently, the keywords and sentiments analyzed within the TCM framework encompass the experiences of surgery or hospitalization and are not restricted to postdischarge transitional care. Therefore, these results should be interpreted with caution. A combination of quantitative and qualitative research methods is essential to gain a comprehensive understanding of patients’ emotional states. Future research can enhance these findings by incorporating focus group interviews with discharged patients and integrating clinical data.

### Limitations

This study had some limitations. First, access to primary data is restricted by South Korea’s Personal Information Protection Act, particularly by the Medical Care Act. Future research can address this limitation by conducting analyses of primary data under strict protocols and anonymization procedures in collaboration with medical research institutions, allowing for the inclusion of detailed clinicodemographic variables. Second, we analyzed posts from patients who shared their health status on the online community. Considering that internet users are likely to have better physical health, information from patients with more severe conditions may have been underrepresented, potentially introducing sample bias. Third, the keywords used as search terms pertained to surgical and inpatient experiences, making it challenging to distinguish between experiences during hospitalization and those during the postdischarge transition period. Fourth, this research classified emotions into only 3 categories. Emotions can be defined in various ways, and we acknowledge the limitations in classifying emotions into more detailed categories due to accuracy concerns. Future research should aim to classify emotions in a more exhaustive manner.

### Conclusions

The sentiment analysis results demonstrated that the KoBERT model had comparable performance levels to those of previous models. For sentiment analysis, the TCM components was categorized into 5 patient-centered domains: health status, care resources, care demand, interaction, and mental state. The sentiment analysis revealed that cardiovascular surgery patients expressed fewer negative sentiments than did cerebrovascular surgery patients, likely because of the relatively simpler procedures generally used in treating cardiovascular conditions. Conversely, patients with cerebrovascular disease who typically required long-term rehabilitation had higher negative sentiment scores. Notably, cerebrovascular surgery patients showed high positive sentiment scores in the “interaction” domain (relating to medical staff) but high negative sentiment scores in the “health status” domain. In contrast, cardiovascular surgery patients exhibited high positive sentiment scores for mental states and high negative sentiment scores for care demands. Collectively, these results suggest that a one-size-fits-all approach is not applicable for managing cardiovascular and cerebrovascular diseases. Instead, considering the differences in sentiment scores for each TCM domain, health care providers should provide personalized patient care during the transition from surgery to postdischarge care.

## Supplementary material

10.2196/65127 Multimedia Appendix 1 A total of 5 domains were classified from the patient’s perspective based on Transitional Care Model (TCM) components, with examples of related terms.

## Abbreviations

AI: artificial intelligence
API: application programming interface
BERT: Bidirectional Encoder Representations from Transformers
KoBERT: Korean Bidirectional Encoder Representations from Transformers
LLM: large language model
ML: machine learning
OECD: Organisation for Economic Cooperation and Development
TCM: Transitional Care Model
