# Supplementary material for: Experience of Cardiovascular and Cerebrovascular Disease Surgery Patients: Sentiment Analysis Using the Korean Bidirectional Encoder Representations from Transformers (KoBERT) Model
Source: JMIR Med Inform. 2025 May 30;13:e65127. doi: 10.2196/65127 (PMC12143735; doi:10.2196/65127)
Supplement: Multimedia Appendix 1 [file medinform-v13-e65127-s001.docx]

Supplemental Table 1. Five domains were classified from the patient’s perspective based on Transitional Care Model (TCM) components, with examples of related terms.

| **Domain** | **TCM Components** | **Description** | **Related Words (examples)** |
| --- | --- | --- | --- |
| Health Status | Screening  Assessing/Managing Risks and Symptoms | The severity of the patient’s health, symptoms, pain, risk of complications, etc. | Ache, symptom, sick, side effect, severe, swelling, discomfort, complication, caution, breathing, health, improvement, hemorrhage, edema, sleep, ICU, headache, healthy, paralysis, rupture, sequelae, inflammation |
| Care Resources | Screening  Collaborating | Caregivers, guardians, financial situation, etc. | Insurance, transfer, caregiver, provide, family, counseling, Mom |
| Care Demand | Screening  Engaging Patients and Caregivers  Educating/Promoting Self-management | Symptoms, self-care, control ability, information acquisition, persistence, etc. | Counseling, medication, care, preparation, control, cause, life, recover, rehab, workout, post-care, disability, |
| Interaction | Staffing  Maintaining Relationships  Engaging Patients and Caregivers  Collaborating | Interactions with medical staff (medical, non-medical, cooperative, uncooperative, etc.) | Teacher, diagnosis, decision, treatment, prescription, professor, nurse, specialist, explanation, reservation, decision, waiting, doctor, hospital |
| Mental State | Promoting Continuity  Fostering Coordination | Anxiety, despair, difficulty, suffering, etc. | Confirm, review, problem, worry, possible, stress, tough, prayer, complex, scary, comfortable, difficult, thankful, prayer, heart, good, tough |
